# Feasibility and Optimization of Donation Advisor: a Decision Support Tool for Deceased Organ Donation and Transplantation

**DOI:** 10.1097/TXD.0000000000001748

**Published:** 2025-02-21

**Authors:** Sonny Dhanani, Rashi Ramchandani, Jill Allan, Natasha Hudek, Christophe L. Herry, Nathan Scales, Neill K.J. Adhikari, Jamie C. Brehaut, Karen E.A. Burns, Michaël Chassé, Akshai M. Iyengar, Maureen O. Meade, Tim Ramsay, Damon C. Scales, Markus Selzner, Alp Sener, Marat Slessarev, Heather Talbot, Matthew J. Weiss, Jeffrey Zaltzman, Andrew J.E. Seely

**Affiliations:** 1 Division of Critical Care, Department of Pediatrics, The Children’s Hospital of Eastern Ontario, University of Ottawa, Ottawa, ON, Canada.; 2 Department of Medicine, Faculty of Medicine University of Ottawa, Ottawa, ON, Canada.; 3 Clinical Epidemiology Program, Ottawa Hospital Research Institute, University of Ottawa, Ottawa, ON, Canada.; 4 Interdepartmental Division of Critical Care Medicine and Institute of Health Policy, Management, and Evaluation, University of Toronto, Toronto, ON, Canada.; 5 School of Epidemiology & Public Health, University of Ottawa, Ottawa, ON, Canada.; 6 Li Ka Shing Knowledge Institute, Toronto, ON, Canada.; 7 Department of Medicine, University of Toronto, Toronto, ON, Canada.; 8 Department of Medicine, Faculty of Medicine, Université de Montréal, Montréal, QC, Canada.; 9 Department of Critical Care Medicine, Queensway Carleton Hospital, Ottawa, ON, Canada.; 10 Department of Medicine, McMaster University, Hamilton, ON, Canada.; 11 Department of Critical Care Medicine, Sunnybrook Health Sciences Centre, Toronto, ON, Canada.; 12 Department of Surgery, University Health Network, Toronto, ON, Canada.; 13 Department of Surgery & Microbiology and Immunology, Schulich School of Medicine & Dentistry, Western University, London, ON, Canada.; 14 Department of Medicine, Western University, London, ON, Canada.; 15 Canadian Donation and Transplant Research Program, Toronto, ON, Canada.; 16 Population Health and Optimal Health Practices Research Unit, Trauma-Emergency-Critical Care Medicine, Université Laval, Quebec City, QC, Canada.; 17 St. Michael’s Hospital, Unity Health Toronto, University of Toronto, Toronto, ON, Canada.; 18 Department of Critical Care, The Ottawa Hospital, Ottawa, ON, Canada.; 19 Division of Thoracic Surgery, Department of Surgery, University of Ottawa, Ottawa, ON, Canada.

## Abstract

**Background.:**

This study aimed to evaluate the ability of Donation Advisor (DA), a validated clinical decision support tool that uses continuous monitoring, variability analysis, and predictive models, to (i) predict likelihood of successful donation after circulatory determination of death (DCD) before withdrawal of life-sustaining measures (WLSM), and (ii) describe ischemia during WLSM in DCD patients.

**Methods.:**

Eligible patients were screened at the 5 sites where DA was implemented. DA reports were generated in real time but shown to clinicians after the donation was complete (noninterventional). Clinicians were interviewed for improvement of the tool.

**Results.:**

We enrolled 34 donor patients in the study; 27 had DCD attempts and 20 proceeded to organ recovery. DA reports were generated before WLSM in all 27 attempted DCD patients (100%) while post-WLSM ischemia reports were generated in 26 of 27 DCD attempts (96%). Nineteen of 34 involved clinicians completed interviews, 10 from intensive care, and 9 from transplantation team members. Following a user-centered design approach, feedback was used to create 5 versions. Revisions included additions, removals, clarifications, and formatting changes; the number of revisions decreased with each amendment. The report’s predictive scores were found to be useful by most practitioners (83%). We identified barriers and drivers to use the report in future practice, some of which may be addressed through improved education and awareness.

**Conclusions.:**

DA can be deployed in real time during the DCD process. The usefulness and usability of the DA report has improved through user feedback; both barriers and drivers to implementation exist.

**KEY POINTS**

**Question**: Is it feasible to design and implement the Donation Advisor (DA) clinical decision support tool to integrate into existing organ donation practices?

**Findings:**  In this 5-center observational study, we deemed it feasible to deploy and incorporate DA into donation after circulatory death processes. Interviews of staff physicians, fellows, and nurses led to 5 iterations that improved the DA tool and identified initial barriers and drivers for the use of DA.

**Meaning:** The usability of DA has been improved before planned clinical integration, which will be evaluated through subsequent multicenter implementation studies.

## INTRODUCTION

Despite its potential to save lives, improve quality of life, and decrease medical costs, thousands of Canadians remain on the growing organ transplant list.^1^ Organ donation after circulatory determination of death (DCD), which involves withdrawing life-sustaining measures (WLSM) after a joint decision between the family and medical team is reached, has increased the number of organs available for transplantation and shortened transplant waiting times.^1^

Unfortunately, organs recovered after DCD suffer from warm ischemic injury that occurs during WLSM, which can make them unsuitable for transplantation.^2,3^ Successful organ recovery is only possible if death occurs within 30 min to 3 h, depending upon the type of organ(s) for donation, after starting WLSM. There are fundamental metrics to predict which donors will die in time to be eligible for donation, but they are not commonly used. This has an impact on (a) donor families who experience undue stress, (b) patients with end-stage organ failure who do not receive organs that are declined due to uncertainty, and (c) healthcare systems through increased time and cost from inefficient organ assessments.^4^ In fact, transplant teams are routinely flown across regions for donor organs only to be declined in at least 30% of cases.^5^ A prediction tool would support the intensive care unit (ICU) physician and donation coordinators to better determine the likelihood of rapid death and therefore successful DCD donation. This would help manage patient families’ expectations and may help identify when not to proceed with DCD.

Using continuously monitored waveform data from a large observational WLSM study, we developed and validated novel models that can predict the likelihood of death within different time limits after WLSM by identifying patterns of reduced heart rate and blood pressure variability during the dying process. We compared this prediction model against models employing previously used clinical parameters, and/or physician judgement. Reduced variability is associated with both increased severity of illness and decreased physiological reserve, and in this case, is associated with more rapid time to death after WLSM.^8-13^ These variability-derived predictive models have the potential to inform clinicians and families on the likelihood of rapid death and organ donation after planned WLSM in intensive care. We have integrated our predictive model into a clinical decision support tool called Donation Advisor (DA). DA uses relevant donor and organ-specific characteristics and continuous waveform data from bedside monitors to (a) provide an updatable (repeatable daily before WLSM) prediction of timing of death after WLSM, and (b) quantify the duration and severity of organ ischemia post-WLSM. The integration of DA into the complex DCD system workflow assists with the objective and continuous assessment of donor and organ suitability pre- and during-WLSM.

While it is the aim of the DA tool itself to predict likelihood of successful donation after DCD, as well as describe ischemia during DCD, the objective of this study was to complete an observational feasibility implementation study and was not powered to evaluate the tool’s outcomes. This study sought to evaluate the clinical and technical feasibility of the DA tool. The secondary objective was to improve the tool based on structured feedback from interviews with healthcare providers and identify barriers and drivers to future implementation.

## MATERIALS AND METHODS

### Development of the DA Tool

DA was developed after prior work combining waveform-derived, clinical and physician predictive models into a single tool.^7,8,14,15^ The predictive model was derived as previously described but using only the 206 Canadian DePPaRT (Death Prediction and Physiology after Removal of Therapy Study) patients with usable data,^9,7^ to account for the variable performance of the clinician’s prediction in this cohort. It includes variability, and additional clinical features, including the peak inspiratory and positive end expiratory pressures, Glasgow Coma Scale, gag and cough reflexes, pH, FiO2, Apache II Score, lactate, spontaneous breathing rate, pupillary reflex, Pco_2_, positive end expiratory pressure, the total ranked circulatory, analgesic, and sedative doses, as well as the physician’s prediction of time to death and confidence. For each patient, the tool generates calibrated probabilities of dying after WLSM, in 15-min intervals (up to 4 h after WLSM), using models employing variability, clinical features, and/or physician prediction. The random survival forest-based prediction models used the C++ implementation of the ranger package with a Python (3.9) wrapper.^16^ The DA software was implemented on Windows-based laptops, generating reports in real time throughout the DCD process, at each site.

For the organ assessment part of the tool, after WLSM, continuous hemodynamic vital sign and oxygen saturation data from patients are used to characterize warm ischemia severity by quantifying the duration spent below multiple thresholds of arterial blood pressure (ABP) and oxygen saturation, and thereby assess the viability of transplanted organs.^4^ Standardized assessment, improved prediction and objective ischemia measurement are hypothesized to help with donation and transplantation decision making and communication with families.

### Continuous Monitoring and Data Collection

To collect the physiologic variability data for the tool, study personnel connected a laptop to the vital signs monitor to capture physiological waveforms (**Figure S1, SDC,** http://links.lww.com/TXD/A726), including the electrocardiogram (500 Hz), invasive ABP (125 Hz), pulse oximeter plethysmography (125 Hz), and respiratory (62.5 Hz) waveforms. For each pre-WLSM report, vital signs waveform data were collected for at least 30 min. The R-peak interval, systolic, diastolic, and mean blood pressure time series were extracted beat-by-beat from the electrocardiogram and ABP waveforms. Seventeen variability metrics were calculated using windows of 750 beats, using up to 60 min of data for each prediction.

**FIGURE 1.**
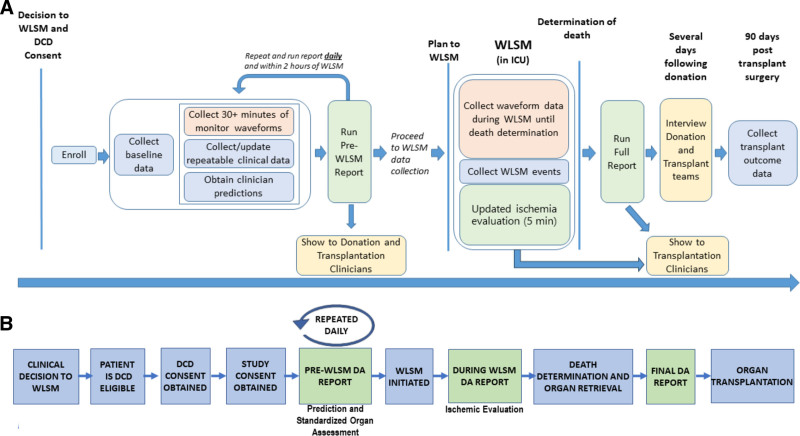
Study workflow of the DA tool. A, Diagram detailing study data collection. B, Diagram detailing the prescribed use of the DA tool within the DCD workflow. DA, Donation Advisor; DCD, donation after circulatory death.

Local research coordinators entered data into the DA tool (**Figure S1, SDC,** http://links.lww.com/TXD/A726), beginning at the time of DCD consent (Figure 1). Data elements included donor demographics, ICU admission diagnosis, medical and social history, circulatory and ventilatory supports, sedative and analgesic dosing, neurological assessments, laboratory results, and organ-specific data, including findings from radiographic imaging. We asked the most responsible physician to predict whether death would occur within 0.5, 1, 2, 3, 6, or >6 h of WLSM, and to rank their confidence (low, moderate, or high). These variables were updated daily, and within 2 h before WLSM, to provide up-to-date DA reports.

### DA Reports

DA reports contained the probability of death within 30, 60, or 120 min after WLSM (**Figure S2a, SDC,** http://links.lww.com/TXD/A726), and clinical details pertinent to DCD suitability and success. For post-WLSM reports, vital signs waveform data, collected from the time of WLSM to the determination of death, were combined with details of how life-supporting measures were withdrawn, to generate assessments of ischemia after WLSM (**Figure S2b, SDC,** http://links.lww.com/TXD/A726). Only the final DA reports from patients who had DCD attempts were shown to the interviewees, several days after the DCD attempt. Practitioners did not have access to reports in real time.

### Participants and Recruitment

#### Ethics

Research Ethics Board approval was obtained from the Clinical Trials Ontario (CTO ID 2117, Implementation of Donation Advisor, a personalized clinical decision support tool for improved efficiency and effectiveness of deceased organ donation and transplantation, initial approval June 10, 2020). Procedures were followed in accordance with the ethical standards of the responsible committee on human experimentation (institutional or regional) and with the Helsinki Declaration of 1975. Consent was waived for study participation of donor and transplant patients. Substitute decision-makers were provided with a description of the study and could opt out of participation at any time. Consent was obtained from healthcare professionals before the interview sessions.

#### Sample Size

For this feasibility study, we did not power for statistical significance, and sample size was not calculated.

#### Sites and Patients

DA was implemented at 5 sites (7 adult ICUs) in Ontario, Canada. An additional site participated solely as a transplant center. Screening occurred in the ICU at each donor site for patients who were medically eligible and consented to DCD.

#### Clinicians

Practitioners from the ICU, donation, and transplantation teams were interviewed from sites at which DA was implemented.

### DA Tool Evaluation

We used 2 complementary frameworks to guide interviews with healthcare providers: User-centered design (UCD), and French’s approach for theory-informed intervention development.^17–19^ The interview guide included 3 sections: (a) introductory demographic questions, (b) “think aloud” review of the DA report focusing on the design, usefulness, and usability of the information in the report, and (c) barriers and drivers to implementation, including questions about whether/how clinicians would prefer to use the tool (**Supplementary Material 1, SDC,** http://links.lww.com/TXD/A726).

### Interview Procedure

Interviewees were sent an initial email invitation to participate and up to 2 reminders for nonresponders at 1-wk intervals. Virtual interviews were audio recorded (Microsoft Teams). A psychologist (>10 y of experience) conducted all interviews, with another team member present to facilitate note-taking. On the rare occasion when only 1 interviewer was present, field notes were recorded and later supplemented from audio recordings.

### Interview Data Analysis

#### User-Centered Design (UCD)

UCD-related comments were categorized into one of 4 broad categories of feedback to improve usability and decision-making (additions, removals, clarifications, and formatting). Categorizations for the UCD portion of the interviews were done inductively by a single rater and reviewed by the study team. Changes to the DA report were made iteratively; 2–3 interviews per iteration of the DA tool were deemed sufficient to identify actionable improvements before the next iteration. This process was repeated until no new major suggestions were noted. Changes to the tool were based on team consensus, in consultation with the steering committee, after review of UCD feedback.

#### Barriers and Drivers to Implementation

Coding for barriers and drivers to implementation was done inductively from interview notes. A single coder developed themes and subthemes, with each new interview, which were revised periodically by study team members and approved by the steering committee.

## RESULTS

### Patients

**FIGURE 2.**
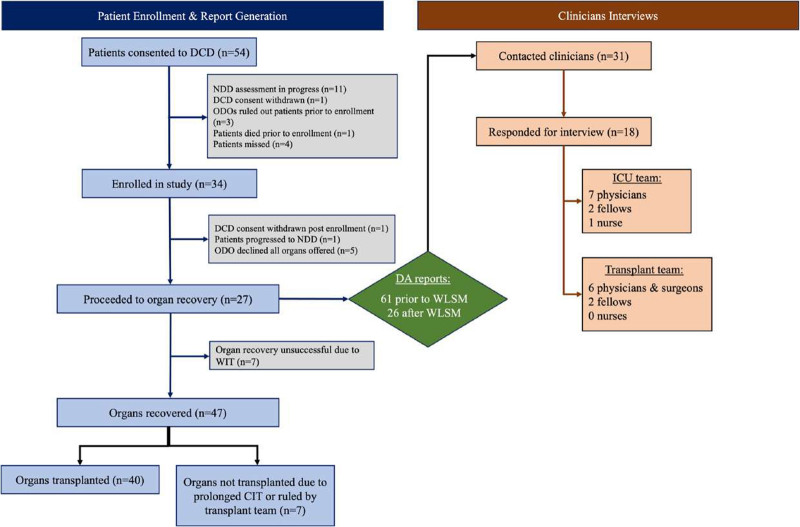
Diagram showing screening and recruitment process for DA report generation, patient, and clinician recruitment. DA, Donation Advisor.

Thirty-four potential donors were enrolled in the study between August 2021 and June 2023. One substitute decision-maker withdrew DCD consent, 1 patient was determined dead by neurologic criteria, and 5 were deemed not eligible for DCD by the Organ Donation Organization. The remaining 27 patients had DCD attempted; the mean (SD) age was 52 (11) y and 11 (41%) were female. Seven patients did not progress to organ recovery due to prolonged time to death following WLSM. Twenty DCD patients had ≥1 organ recovered, for a total of 40 organs recovered and transplanted. Thirty-five of the organs were transplanted at transplant centers participating in this study (25 kidneys, 2 livers, 1 pancreas, and 7 double lungs). Five organs were transplanted at nonstudy centers and were lost to follow-up. Figure 2 summarizes the patient and clinician recruitment process.

### DA Reports

Recordings were performed over a median of 2 consecutive days (range 1–4), spanning a median time of 27 h (range 0.5–77.5). Sixty-one DA reports, with a median recording duration of 30.6 min (range 30–85), were generated for serial assessments before WLSM for prediction of time to death, and 26 DA reports, with a median recording duration of 51 min (range 17–347) were generated during WLSM for organ ischemia assessment.

### Feasibility

DA prediction reports were generated multiple times before WLSM to refresh prediction information. Reports were successfully generated before WLSM in less than 2 min in all 27 patients, with 24 of 27 (89%) patients having at least 2 prediction reports, an initial and an updated version (see Table 1). Post-WLSM ischemia reports were successfully generated in 26 of 27 DCD attempts (96%); in 1 case the research team was not notified when WLSM occurred.

**TABLE 1.** Feasibility summary of DA implementation

Variable	Attempted DCD (n = 27)	Successful DCD (n = 20)	Unsuccessful DCD (n = 7)
Clinical features			
Sex (male)	16/27 (59%)	12/20 (60.0%)	4/7 (57.0%)
Age (y)	57.0 (29.0–66.0)	52.5 (29.0–66.0)	59.0 (33.0–64.0)
MAP (mm Hg)	83.0 (72.0–126.0)	81.5 (72.0–126.0)	86.0 (78.0–102.0)
HR	67 (51.0–106.0)	69.5 (51.0–106.0)	66.0 (63.0–102.0)
APACHE II score	24.0 (14.0–36.0)	25.0 (14.0–36.0)	20.0 (16.0–29.0)
GCS	4.0 (3.0–10.0)	3.5 (3.0–10.0)	5.0 (3.0–9.0)
FiO2 (%)	30.0 (25.0–100.0)	30.0 (25.0–100.0)	30.0 (25.0–40.0)
Spontaneously breathing	16/19 (84.0%) n = 19	11/14 (79.0%) n = 14	5/5 (100.0%) n = 5
Gag absent	17/20 (85.0%) n = 20	13/16 (81.0%) n = 16	4/4 (100.0%) n = 4
Cough absent	14/27 (52.0%)	11/20 (55.0%)	3/7 (43%)
Pupillary reflex absent	10/21 (48%) n = 21	8/16 (50%) n = 16	2/5 (40%) n = 5
On pressors/inotropes	5/27 (19.0%)	5/20 (25.0%)	0/7 (0%)
Time to death (min)	36.0 (11.0–7000.0)	24.5 (11.0–153.0)	1002.0 (330.0–7000.0)
Variability features			
DBP Poincaré SD2	1.82 (0.59–10.05)	1.67 (0.59–10.05)	2.22 (1.05–2.91)
DBP DFA AUC	0.5 (–0.46 to 1.52)	0.47 (–0.46 to 1.52)	0.62 (0.19–0.91)
SBP DFA Alpha 1	0.75 (0.13–1.3) n = 23	0.81 (0.13–1.3) n = 18	0.44 (0.23–0.79) n = 5
RRI Grid count	1.8 (0.2–5.68) n = 23	1.58 (0.2–5.49) n = 18	3.34 (0.64–5.68) n = 5
Feasibility outcomes			
Number of pre-WLSM reports	61	42	19
Number of post-WLSM reports	26	19	7
Fraction of patients with pre-WLSM reports	27/27 (100%)	20/20 (100%)	7/7 (100%)
Fraction of patients with post-WLSM reports	26/27 (96%)	19/20 (95%)	7/7 (100%)
Number of pre-WLSM reports/patient	2 (1–4)	2 (1–3)	3 (1–4)
Pre-WLSM recording analysis duration (s)	39 (15–103)	40 (15–103)	34 (15–49)
Post-WLSM recording analysis duration (s)	4 (2–12)	4 (3–11)	3 (2–12)
Pre-WLSM report generation and review (s)	97 (14–827)	130 (14–827)	79 (18–407)
Post-WLSM report generation and review (s)	46 (11–557)	54 (11–557)	31 (12–296)

Demographics of attempted DCD patients and feasibility outcomes, separated by organ donation status. Continuous variables are presented as median (range), while categorical variables are presented as fraction (%). The Grid Count sums the total over all pixels, divided by the number of pixels in the grid. Also known as Box Count.

DBP, diastolic blood pressure; DFA AUC, the area under the curve of the log-log plot of the fluctuations over increasing time scales for the detrended integrated series; DFA Alpha 1, the slope on the log-log plot of the fluctuations over increasing time scales for the detrended integrated series; FiO2, fraction of inspired oxygen; GCS, Glasgow Coma Scale; Grid Count, The time series is transformed into a grid, similar to a discretized Poincaré plot, with each pixel assigned a 1 if it was visited during the time series, and 0 otherwise; HR, heart rate; MAP, mean arterial pressure; Poincaré SD2, the SD of the Poincaré plot along the line of identity; RRI, R-peak to R-peak interval; SBP, systolic blood pressure.

### Time to Death Predictions

We were not powered to assess model performance (**Figure S3, SDC,** http://links.lww.com/TXD/A726) in this feasibility study. For descriptive purposes, the mean probability of dying within 30 min predicted by DA was 47% (95% confidence interval [CI], 38%-56%), while the actual frequency of dying in 30 min was 45% (95% CI, 26%-63%). At 60 min, the DA-predicted probability of dying was 62% (95% CI, 0.53-0.72), while the actual frequency was 71% (95% CI, 0.52-0.89). Finally, at 120 min, the mean predicted probability of dying was 75% (95% CI, 68%-83%), while the actual frequency of dying in 120 min was 71% (95% CI, 52%-89%). Using the clinician’s most recent prediction before WLSM, 59% (16/27) of clinicians correctly predicted death within 2 h (66% for death within 30 min, 63% for death within 1 h, and 70% for death within 3 and 6 h).

Predictions for most patients remained stable over multiple assessments. The model combining all feature types tended to be more stable over time, with a median value for the maximum observed probability change of 0.02, compared with 0.12 and 0.11 for models using clinical or variability features alone, respectively. Four patients demonstrated probability changes in the combined model for death within 2 h of >0.4, 2 of which improved prediction (**Figure S4, SDC,** http://links.lww.com/TXD/A726).

### Clinician Interviews

Overall, 19 of 34 contacted practitioners participated in interviews, which included 10 ICU team members (7 physicians, 2 fellows, and 1 nurse) and 9 transplant team members (4 nephrologists, 2 fellows, and 3 surgeons). Five interviewees were female. Years of ICU/transplant experience averaged 12 y (range 1–4). Interviewees were affiliated with The Ottawa Hospital (n = 12), the Queensway Carleton Hospital (n = 1), University Health Network (n = 4), and London Health Sciences Centre (n = 2). The average interview duration was 33 min (range 17–50).

### Iterations of DA Report

**FIGURE 3.**
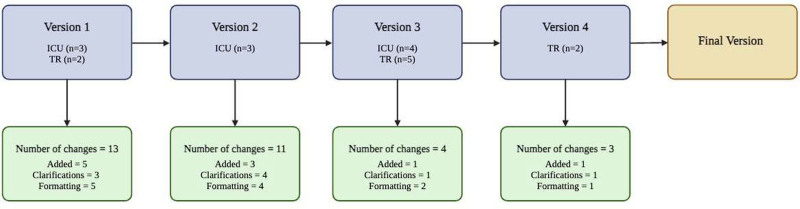
Flow diagram outlining the iterative change process for each version of the DA report from feedback provided by ICU and TR team members (n = 19). DA, Donation Advisor; ICU, intensive care unit; TR, transplant.

The initial DA report underwent 5 versions with 4 major changes based on interview feedback (see Figure 3). Changes made to the report included instances where information was as follows: (a) added, (ie, adding the time delta from WLSM to death); (b) removed (ie, omitting several graphs from the ischemia section); (c) clarified, (ie, when visuals were added to display relationships between predictive models); and (d) re-formatted (ie, when related data was grouped together, as in the case of vital signs and circulatory support, or respiratory data and ventilatory support).

The most meaningful change to the report was (a) designing 2 separate user-specific reports for the donation and transplant teams. Other prominent changes included (b) moving the prediction model to the first page, (c) reducing the page count from 10 to 8 pages, (d) color coding and reducing the number of graphs in the ischemia report, and (e) tailoring organ assessment reports to include decisive information.

### Evaluation of DA Tool Implementation

Table 2 provides an overview of 17 major themes identified during interviews. Notable barriers to tool use included a desire for a better understanding of the tools’ validation and predictive strengths (n = 9), and accessibility in real time before/during WLSM (n = 9). The most frequently reported drivers included the potential to conserve personnel and time resources (n = 11), and to improve communication among healthcare providers (n = 6). When asked how long clinicians would spend looking at the DA report, most respondents (n = 7) indicated they would spend 5–10 min; with others indicating as little as one (n = 4) or as much as 20 min throughout the WLSM process (n = 1).

**TABLE 2.** Themes related to implementation of the DA tool identified through interviews ICU and transplant team members (n = 19)

Theme	Description	ICU (n = 10)	Transplant (n = 9)	Total (n = 19)
Barriers				
Validation/predictive strength	Uncertainty around the tool validation and predictive strength in probability of time to death may prevent use	5 (50%)	4 (44%)	9 (47%)
Tool and data access	Challenges with tool and data access required to use the tool may prevent use, who should be able to access the tool and how it will be accessed	3 (30%)	6 (67%)	9 (47%)
Education	The expressed need for education around the derivation, validation, and predictive strength may prevent use	3 (30%)	3 (33%)	6 (32%)
Document size	Large document size may prevent use, particularly in the context of other donor information received from other sources	1 (10%)	5 (56%)	6 (32%)
Ethics, roles, conflict of interest	Physician concern over their roles and responsibilities in the organ donation process may discourage use of tool	4 (40%)	0	4 (21%)
Tool vs physician gestalt	Physicians may prefer to use their own gestalt over predictive tools	2 (20%)	1 (11%)	3 (16%)
Experience with other prediction tools	Poor performance of previous tools may negatively impact physician interest and willingness to use new prediction tools	1 (10%)	2 (22%)	3 (16%)
Creating routines	Challenges with incorporating tool into current routines to increase familiarity and use	2 (20%)	1 (11%)	3 (16%)
Workflow	Personnel needs around who will collect data and produce reports may prevent generating reports	2 (20%)	0	2 (11%)
Understanding how to use predictions in practice	Uncertainty around how to use the predictive model values in practice may decrease use	0	2 (22%)	2 (11%)
Resources	Potential for increased need of monitoring equipment and other resources to complete DA reports may prevent ability to use in practice	1 (10%)	0	1 (5%)
Procurement program protocol	Institutional protocols may affect ability to use ischemia component in practice	0	1 (11%)	1 (5%)
Drivers				
Resources	Potential for improved allocation resources (financial, human resources, time) in relation to dispatching procurement teams	3 (30%)	8 (89%)	11 (58%)
Communication among healthcare providers	Tool may improve communication among healthcare providers, both the ICU and transplant side, via graphical data and summaries that can be discussed remotely	3 (30%)	3 (33%)	6 (32%)
Recipient outcomes and care	Tool may facilitate improved recipient outcomes and care (eg, early implementation of delayed graft function protocols)	0	4 (44%)	4 (21%)
Optimism	Feelings of optimism and excitement about predictive value of tool may increase use	2 (20%)	2 (22%)	4 (21%)
Validation/predictive strength	Confidence in the tool validation and predictive strength in probability of time to death may encourage use	2 (20%)	2 (22%)	4 (21%)
Tool vs physician gestalt	Physicians may place more confidence in the predictive tool than their own judgment, encouraging its use	2 (20%)	1 (11%)	3 (16%)
Organ wastage	Use of tool may decrease number of organs considered for transplantation, but ultimately not transplanted	0	3 (33%)	3 (16%)
Confidence	Using tool may increase physician confidence in the DCD process	0	1 (11%)	1 (5%)

DA, Donation Advisor; DCD, donation after circulatory death; ICU, intensive care unit.

When asked when they would prefer to view the DA report, most ICU team respondents indicated a preference to see the report after the decision to proceed to WLSM (n = 5), after family has consented to donation (n = 2), or before approaching donation coordinators (n = 1). Conversely, transplant team respondents often indicated wanting to see the report multiple times, including at the time of the organ offer (n = 6), just before sending a recovery team (n = 1), after a notable change in the patient’s clinical status (n = 1), or in real time during WLSM (n = 2). One transplant clinician indicated wanting to see the report only after WLSM (n = 1).

Most practitioners indicated the usefulness (n = 15) of the DA tool if used in practice, with the predictive model (n = 6), the ischemia report (n = 2), or the organized patient overview (n = 2) being the most helpful. Four respondents were unsure of how to use the DA tool (n = 2) or did not foresee themselves using it (n = 2) in their role. Over half of the clinicians found the predictive ability of the DA report useful to set expectations and assist with how and when to approach families, thus achieving its intended function. When asked if they would use the tool in practice most respondents indicated that they would (n = 12), 3 were uncertain but still willing to utilize, 2 responses were unclear, and 2 respondents indicated they did not plan to use DA because their current healthcare model was to accept all potential donors regardless of prediction of time to death.

When asked what decisions the DA report might help with, several indicated it would help determine donor suitability (n = 10) or organ suitability (n = 7); some indicated the DA report could guide conversations or communication with patients’ families (n = 11), assist with end-of-life care planning (n = 3), time-management in the ICU (n = 2), decide whether an organ recovery team should be dispatched (n = 6), and guide transplant aftercare for recipients (n = 2).

## DISCUSSION

This is the first study of DA, a novel monitoring-based variability-derived software tool designed to improve the prediction of time to death after WLSM in ICU and to assess the duration and severity of ischemia for donated organs, all to assist donation and transplantation decision-making. In this observational feasibility implementation study, we successfully generated and delivered the DA clinical decision tool in 5 donation sites with rapid successful generation of predictions and reports in all cases. We were not powered to assess model performance, but the model demonstrated decent calibration at the population level. In future studies, more rigorous assessments of model calibration and discrimination performance will be possible.

We improved the useability and usefulness of the DA report from a UCD perspective. Clinician interviews led to feedback resulting in 4 progressive iterations, with 2 major lessons emerging. First, healthcare provider interviews revealed majority support of the DA report for future clinical use in real time. Second, donation and transplant clinicians would require separate specific reports. The number and extent of changes decreased with each iteration of the report, as would be expected with a UCD approach, indicating the usability and usefulness of the tool increased with each iteration. Future implementation work will consider role-specific reports, potentially taking advantage of the dynamic features of online applications. Similar methods have been widely used to develop decision-support tools for both patients and healthcare providers.^20,21^ The use of any clinical decision support depends on the trust of the clinicians and their interpretation of the results of the tool; therefore, we aim to evaluate this directly in the next step of evaluation, namely, interventional studies.

One of the noted drivers to using the tool included communicating with patients’ families around end-of-life care and time to death. Families who consent to DCD but whose loved ones did not go on to donate report feeling “a waste of precious life-giving organs and hospital resources” and a need for comprehensive support services to cope.^4^ Over half of clinicians found the predictive ability of the DA report useful to assist with family conversations, such as when/how to approach families and setting expectations. Krmpotic et al found that nearly half of families consented to organ donation when approached for DCD, but only 57% of these were able to donate an organ.^5^ The uncertainty surrounding donation outcomes has been shown to add stress for families and is hypothesized to be a reason for declining organ donation and DCD consent rates.^5^ The impact of missed donation opportunities also impacts healthcare providers and may lead to burnout.^22^

In this study research coordinators administered the tool; while it is too early to say how best to implement this tool in diverse jurisdictions, our current belief is that organ donation organizations are optimally suited to implement the interventional tool. We believe that a larger interventional study is required to further evaluate physician confidence as well as provide evidence as to the performance of the predictive model in a diverse population and real-time environment.

This study has several limitations, principally relating to its observational nature, and the small number of patients and hospital sites in a single region. Importantly, we did not offer the outputs of the DA tool in real-time during donation opportunities but sought feedback only after the clinical case was completed, to prevent the tool from influencing decision-making during its development phase. Future interventional studies of real-time deployment for decision-making may reveal different utility or barriers when seeing the tool at the bedside during the donation and transplantation decision-making process. For interviews we only used a single coder; however, UCD methods do not require transcripts or double coding. We did not address whether the device is better than clinical judgment or better at assessing ischemia in otherwise previously viable organs. In future studies, where we can randomize centers and or patients to the intervention, we can evaluate the degree of value added to both clinical and financial outcomes.

## CONCLUSION

This study was an early step in the development and implementation of the DA clinical decision support tool. Using a UCD approach, feedback gathered from practitioners was applied to iteratively improve the usefulness and usability of the DA report. Several barriers and drivers to using the tool in practice were identified, which will be further explored using structured theory-based methods in future work. A large-scale prospective study is needed to evaluate the interventional implementation of DA, analyze clinical impressions in real-time use, and to further validate the DA tool’s predictive ability and assessment of ischemia in organs for donation.

## ACKNOWLEDGMENTS

We would like to acknowledge Health Canada and PSI for funding as well as the Trillium Gift of Life Network (TLGN), Canadian Critical Care Trials Group (CCCTG), Canadian Donation and Transplantation Research Program (CDTRP), The Ottawa Hospital (Civic and General Campuses), Kingston Health Sciences Centre, London Health Sciences Centre (University and Victoria Hospitals), Sunnybrook Health Sciences Centre, and Queensway Carleton Hospital for their collaboration and support toward this study.

## Supplementary Material


