# Supplementary material for: Feasibility and Optimization of Donation Advisor: a Decision Support Tool for Deceased Organ Donation and Transplantation
Source: Transplant Direct. 2025 Feb 21;11(3):e1748. doi: 10.1097/TXD.0000000000001748 (PMC11850036; doi:10.1097/TXD.0000000000001748)
Supplement: Supplementary file 1 [file txd-11-e1748-s001.pdf]

Supplementary Materials

Supplementary Materials: Interview guide used with questions centered around gauging clinicians' opinions of the DA tool.

Date: ______________

Time: ______________

Moderator: ______________

Interview Guide:

Think Aloud Protocol Evaluation of Donation Advisor Tool

SET UP

- Set up a 30-minute teleconference with the clinician
- Written informed consent document should be send to them prior to the session
- Ensure that the interviewee is emailed a de-identified PDF file including the DA-Pre Report that was run immediately prior to withdrawal of life support measures (WLSM) or the DA-Full Report that was run immediately post pronouncement of death.
- When contacting the interviewee for the interview, the interviewee's name will only be recorded on a master interviewee list. Any record of responses to the questions in this guide will only identify the interviewee by their Participant ID number.
- Depending on the report(s) being reviewed (Pre vs Full) the interviewer will adjust the instructions accordingly.

INTRODUCTION SCRIPT FOR MODERATOR

*Thank you for meeting with us. If you recall from our email, we are developing a novel tool called **Donation Advisor (DA)**, a clinical decision support instrument designed to assist in identifying successful donors after circulatory determined death (DCD). Our overall goal is to use this tool to improve the efficiency of the donation process after DCD. We have Health Canada and PSI funding to implement DA. Our discussion should take 20 to 30 minutes.*

We are currently assessing the DA tool and its user interface, and would like to get your opinions on the tool itself, whether you would or wouldn't use it in your practice, barriers and drivers to its use, etc. To that end, we have computed the DA report for

- *(where the interviewee was not involved in the DCD case) a DCD eligible patient*
- *(where the interviewee was involved in the DCD case) a recent DCD eligible case that you were involved in, and that information is presented in the PDF file we sent you. Please note there is no identifying patient information on it. Have you received it? Can you pull it up on your computer now?*

Great. Before we get started, we should mention a few things:

- *<u>We sent you an informed consent</u> document by email. Have you read through the consent? Do you have any questions? Do you give consent for us to use your responses as part of our study?*

- *<u>We are not clinicians</u>, so please bear with us if we need to ask you to clarify any clinical language.*
- *<u>You can decide you'd rather not answer any particular question.</u> We'd like you to answer these questions as truthfully and completely as possible, however, you can skip any questions that make you uncomfortable.*
- *<u>Everything you say is confidential.</u> Your name will not be mentioned in any report, your comments will only ever be reported anonymously.*
- *<u>We appreciate your suggestions.</u> Throughout the session, I'll be asking you to tell us what you are thinking about the different questions. We'll greatly appreciate any thoughts you might have about how to improve the tool.*
- *<u>We are recording this session by audiotape.</u> This will help ensure we learn as much as possible from these sessions. Is this OK with you? Recordings will be kept until the end of the study and then will be destroyed.*

Any questions so far?

First, we need some information about you.

<u>Participant Data</u>

1. What institution do you work at:

   - ☐ The Ottawa Hospital - General and/or Civic Campuses
   - ☐ Kingston General Hospital
   - ☐ London Health Science Centre University and/or Victoria Hospital
   - ☐ St. Michael's Hospital
   - ☐ Sunnybrook Hospital
   - ☐ Hamilton Health Sciences
   - ☐ University Health Network
   - ☐ Queensway Carleton Hospital
   - ☐ Trillium Gift of Life Network (TGLN)

2. What is your professional role?

   ☐ RN
   · How many years of nursing practice: _____
   · How many years of ICU nursing practice: ______

   ☐ TGLN
   · Role: ________________
   · How many years of TGLN practice: ____

   ☐ ICU Fellow
   · Year 1 or 2? ____

   ☐ Attending Physician
   · Years of clinical practice in ICU: ____

   ☐ Transplant Surgeons
   · Years of clinical practice in transplant surgery: ___
   · Specialty (i.e., organ(s) of interest): ____________________

☐ Transplant Fellow

· Year 1 or 2? ___

· Specialty (i.e., organ(s) of interest): ____________

☐ Other (specify): ______________________

· Area of Expertise: ___________________

Remind QHC voluntary

3. How would you describe your gender identity (categories for prompting and data capture only)?

o man/male o woman/female o transgender man/male o transgender woman/female

o non-binary o prefer to self-identify: ___________________ o prefer not to answer

OK, let's begin the main part of the session.
I would like to draw your attention to the PDF file of the Donation Advisor reports of

- *(where the interviewee was not involved in the DCD case) a DCD eligible patient; OR*
- *(where the interviewee was involved in the DCD case) a recent DCD eligible case that you were involved in,*

The donation advisor or DA report is intended to be a tool used by critical care and transplant physicians to be able to better predict likelihood of successful organ retrieval following DCD, as well as to objectively characterize the degree of ischemia of the organs retrieved post DCD. Both are based on inputs from vital sign waveforms.

Today, we will be focusing on the <Pre-WLSM report or Full report as applicable>.
<If reviewing the pre-WLSM report> There are two components to this report. The first component (the first 2 pages of the report) focuses on the predictive tool. This is a repeatable assessment (i.e. May be performed several times, with the last one immediately prior to withdrawal of life-sustaining measures). The second component (starting on page 3) provides assessment of the individual organ function prior to WLSM <if the interviewee is from the transplant team, direct them to review the organ of their focus. If the interviewee is from the donation side, explain that this section is directed toward the transplant teams>

<if reviewing the Full report> There are three components to this report. The first component (the first 2 pages of the report) focuses on the predictive tool. This is a repeatable assessment (i.e. May be performed several times, with the last one immediately prior to withdrawal of life-sustaining measures). The second component of the report on page 3 is an ischemia evaluation tool. The third component (starting on page 4) provides assessment of the individual organ function prior to WLSM <if the interviewee is from the transplant team, direct them to review the organ of their focus. If the interviewee is from the donation side, explain that this section is directed toward the transplant teams>

What we'd like you to do is look through this information as <u>if you are currently involved in the organ donation/transplantation process for this person.</u> I'd like to have you read through and interact with this tool as you think you would in your daily practice.

The one difference is that we DON'T want you to do it quietly. In fact, we would like you to "talk aloud" and verbalize anything that you are thinking to yourself. The main purpose of this exercise is to help us understand what you are thinking about the tool, various aspects of the display, how it agrees or disagrees with your

decision, etc. So it's very important that you verbalize everything you read and everything you think about as you are reading. So for example… ***(Demonstrate)***

Because we are audiotaping this, if I don't hear anything from you in a while, I'll remind you to continue talking.

Once you have worked through the information in the tool to your satisfaction, just let me know that you are done; at the end I have a few specific questions I'd like to ask you about the tool.

Do you have any questions?

Ok…let's get started.

- Encourage the interviewee to review first the DA-Pre Report to inform their decision. Such as
  - (For interviewee's who are donation physicians) about whether this person will be a successful or unsuccessful candidate organ donation, or
- Second to review the DA-Full Report (especially for interviewee's who are a transplant surgeons) to review the DA-Full report (to consider organ transplantation).

Possible Prompts

- 'what is this tool telling you about the decision whether to recommend this person for organ donation?'
- '*(where the interviewee was involved in the DCD case)*
  - 'do you recognize the case?'
  - 'Is this information consistent with what you remember about the patient?'

Participant ID ___________

INCIDENT LOG SHEET – To be completed by the Note Taker while the participant works through the mock survey. Incident interview questions (based on the incidents that were flagged) will be asked at the end of the survey walkthrough session.

Time at Start of Session: ____________

**Page number**	**Question (# or description)**	***INCIDENTS***			**Follow up?**	**Incident Follow up with Participant**<br><br>***(I noticed that when you reviewed page X, X happened. Can you tell me what happened?)***
	**Circle data element with issue/incident**	**Hesitation?**	**Request for help?**	**Descriptions of issues/incidents**	Check if yes<br>✓	
**1**	**Demographics**<br>Age:<br>Sex/Gender<br>Height<br>Weight<br>BMI<br><br>**ICU Admission**<br>RFA<br>ICU Admission Date<br>LOS<br>Cardiac Arrest<br>Cardiac Arrest Duration<br>CPR Duration<br><br>**Past Medical History**<br><br>**Diagnoses during hospitalization**					

Participant ID ___________

Page numbe r	Question (# or description)	INCIDENTS			Follow up?	Incident Follow up with Participant<br>***(I noticed that when you reviewed page X, X happened. Can you tell me what happened?)***
	**Circle data element with issue/incident**	**Hesitation ?**	**Request for help?**	**Descriptions of issues/incidents**	Check if yes<br>✓	
	**Social History**<br>Smoking<br>Alcohol<br>Drugs<br><br>**Prediction of Time to Death***<br>Overall<br>Variability-Derived<br>Clinical Model<br>Clinician Prediction					

**Should the interviewee ask for an explanation of the Prediction report, the interviewer can state: 'The Prediction report is based on the predictions obtained from bedside clinicians during the DePPaRT study. The probability of dying at a given time point is an estimate of the fraction of patients that passed away within that time frame and had the same combination of clinician's prediction time and confidence level.' <Note: later in the study, this explanation may include that data obtained during this Donation Advisor Study will be included in this model.>*

2	**Circulatory Support**<br><br>**Vitals**<br><br>SBP DBP MAP<br><br>HR<br><br>SpO2<br><br>Temperature<br><br><br>Inotropes and Vasopressors<br><br>ECMO IABP<br><br>**Ventilatory Support**<br><br>Route<br><br>Duration<br><br>Settings<br><br>Mode FiO2<br><br>Respiratory Rate<br><br>Tidal Volume<br><br>PS PEEP<br><br>Measurements<br><br>PIP MAP<br><br>Spontaneous Respiratory Rate<br><br>Actual Respiratory Rate<br><br>Minute Ventilation<br><br>**ABG Results**<br><br>**Neuro Exam**<br><br>GCS<br><br>Pupillary Reflexes<br><br>Cough Reflex<br><br>Gag Reflex					

Page number	Question (# or description)	INCIDENTS			Follow up?	Incident Follow up with Participant *(I noticed that when you reviewed page X, X happened. Can you tell me what happened?)*
	**Circle data element with issue/incident**	**Hesitation?**	**Request for help?**	**Descriptions of issues/incidents**	Check if yes ✓	
	Corneal Reflex<br>Spontaneous Respiratory Effort<br>Oculovestibular Reflex<br><br>**Analgesics**<br>**Sedatives**<br>**NDD Assessment**<br>Performed?<br>**Labs**<br>WBC Hb Platelets INR<br>Na K Glucose Creatinine<br>Troponin I/T<br>Lactate<br><br>Positive Sputum Gr Stain/Culture<br>Positive Urine Culture<br>Positive Blood Culture					

**DA-Full Report**<br><br>**Page 4**<br><br>**(Transplant Clinicians only)**	**Process of Withdrawal of Life Sustaining Measures**<br><br>**Ischemic Evaluation During WLSM**<br><br>**Graphs****					

*** Should the interviewee ask for an explanation of the Time under X graphs, the interviewer can state: These graphs depict the time spent under specific thresholds (i.e., MAP < 75). Time under any threshold of interest can be viewed on this graph.*

Page numbe r	Question (# or description)	INCIDENTS			Follow up?	Incident Follow up with Participant<br>*(I noticed that when you reviewed page X, X happened. Can you tell me what happened?)*
	Circle data element with issue/incident	Hesitation ?	Request for help?	Descriptions of issues/incidents	Check if yes<br>✓	
**Organ Assess-ment**<br>**DA-Pre Report**<br>**P 3-7),**<br>**DA-Full Report**<br>**P 4-8**	**Organ Assessment**<br>**Kidney**<br>Comorbidities<br>Labs<br>Urine Output<br>Investigations					
	**Organ Assessment**<br>**Liver**<br>Comorbidities/History<br>Labs<br>Investigations					
	**Organ Assessment**<br>**Pancreas**<br>History<br>Insulin<br>Labs<br>Investigations					

Page number	Question (# or description)	INCIDENTS			Follow up?	Incident Follow up with Participant<br>*(I noticed that when you reviewed page X, X happened. Can you tell me what happened?)*
	**Circle data element with issue/incident**	**Hesitation?**	**Request for help?**	**Descriptions of issues/incidents**	Check if yes<br>✓	
	**Organ Assessment**<br>**Lung**<br>ABGs<br>Comorbidities / History<br>Ventilatory Support<br>Settings<br>Measurements<br>Investigations					
	**Organ Assessment**<br>**Heart**<br>Comorbidities/History<br>Circulatory Support<br>Labs<br>Investigations					

Once the participant has finished reviewing Donation Advisor (DA) tool, note the elapsed time (i.e. how much time they spent reviewing and talking about the tool before saying they are 'done':

Elapsed Time: ________________

(Following the Think Aloud part of the interview) We now have few specific questions for you:

DA-Pre report only:

1. Do you find the DA-Pre report useful in your role as part of the donation/transplantation process?

   Prompts: What did you like? What did you not like?

   a) What decisions would it help with? (Did it provide you with useful information, the right information for your decisions?)

   b) Would it help to identify successful candidates? Unsuccessful candidates

   c) Would it help with other decisions? What decisions

DA-Full report only:

2. Do you find the DA-Full Report useful in deciding whether to transplant the organ? (Did it provide you with useful information, the right information for your decisions?)

   Prompts: What do you like? What do you not like?

   a) Does it help with other decisions?

3. In general, do you have any feedback regarding how the data is displayed?

   a. Do the graphs show the data in the way that you would want to see it?

DA-Pre and DA-Full reports

4. Is the tool something you would use in your practice/role to help with donation/transplant decisions?

   Prompts: Why/why not?

5. If you were to use this tool in your practice, how long do you think you would spend examining the tool for each patient?

   Prompts: We need to design this tool for how it will be used; would you use it for 10 seconds, 10 minutes…?

6. **When** would you like to see the report during the DCD decision process?

7. Can you think of important **benefits** that you might anticipate from using this tool in your role in the organ donation/transplant process?

8. What **barriers** might get in the way of a report like this being used in the organ donation/transplantation process?

   Prompts: Factors that would interfere with use of this tool in practice for you or your colleagues.

9. Can you think of important **drivers** we could use to increase use of this tool with you or your colleagues?

   Prompts: Factors that would increase use of this tool in practice?

10. Any additional suggestions?

That's all we have for you; Thank You!

Supplementary Figures

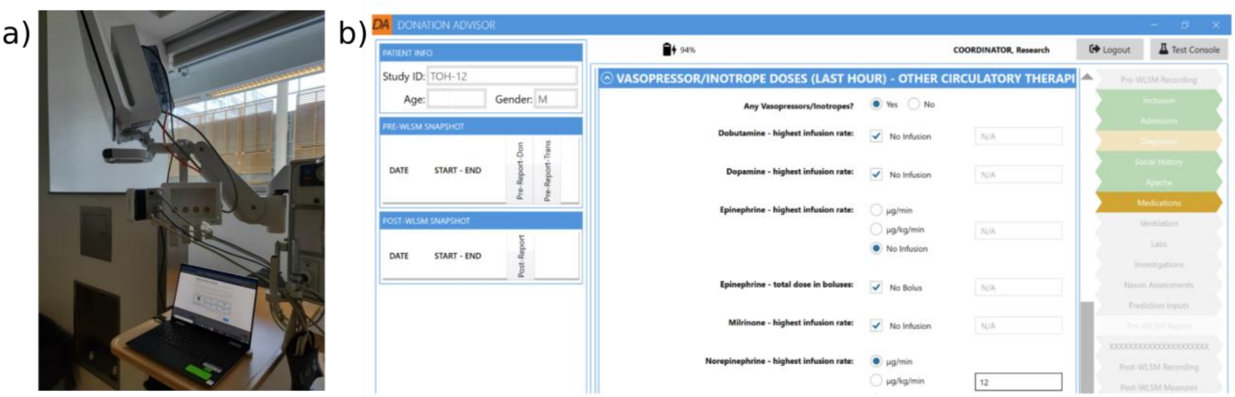

Supplementary Figure 1:Implementation of the DA tool. a) The DA tool was implemented on a Windows laptop, and waveform data was collected via a serial connection to the bedside monitor. b) Example screenshot showing data collection using the DA tool.

a)

Prediction of Time to Death

Prediction Date/Time: 2021-08-29 08:04

Overall Probability of Death*

Probability of Death in % (± SD)	
<30 min	**82** (± 7)
<1 hour	**94** (± 3)
<2 hours	**94** (± 5)

*The overall probability of death is derived from a random survival forest model employing features from all three domains (variability, clinical, and clinician's prediction)

b)

Process of Withdrawal of Life Sustaining Measures

Location	PACU
WLSM Commenced	2021-08-29 09:25
Circulatory Arrest	2021-08-29 09:56
Declaration of Death	2021-08-29 10:01

Time to Death (WLSM→Declaration of Death) ((D) HH:MM)	00:36
Cause of Death	Hemorrhagic Stroke

Ischemic Evaluation During WLSM

Assessed using waveform data captured between 2021-08-29 09:25 and 2021-08-29 10:03

	No Ischemia		Mild Ischemia		Moderate Ischemia		Severe Ischemia		Any Ischemia	
	Reading	(HH:MM)	Reading	(HH:MM)	Reading	(HH:MM)	Reading	(HH:MM)	Reading	(HH:MM)
**SBP**	>90	00:13	75–90	00:04	50–75	00:08	≤50	00:10	≤90	00:23
**MAP**	>75	00:09	60–75	00:06	45–60	00:10	≤45	00:11	≤75	00:27
**SpO_2**	>80	00:19	65–80	00:01	50–65	00:01	≤50	00:15	≤80	00:18

Vital Signs Over Time

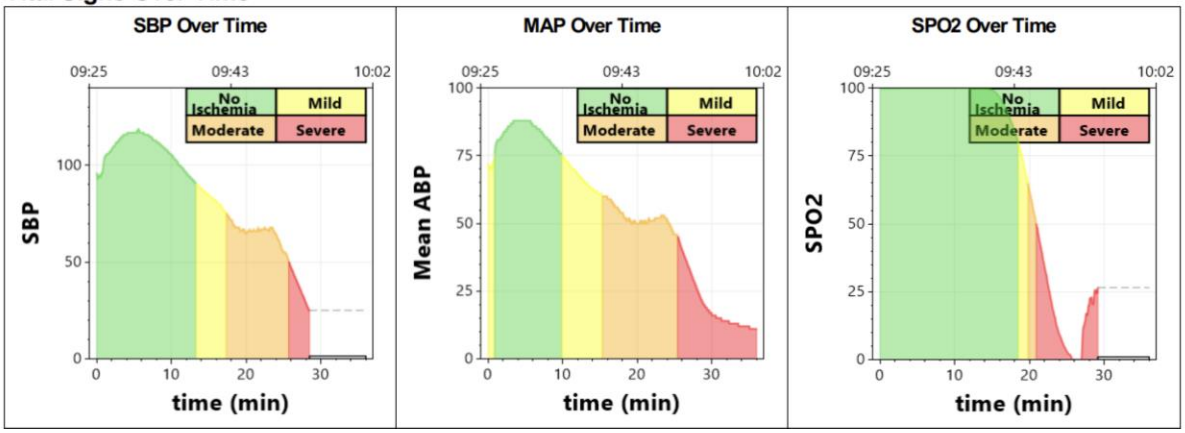

Supplementary Figure 2: Example of a DA pre-WLSM time to death prediction (a) and post-WLSM ischemia evaluation (b) evaluation of life sustaining measure withdrawal showing ischemia evaluation and vital signs over time

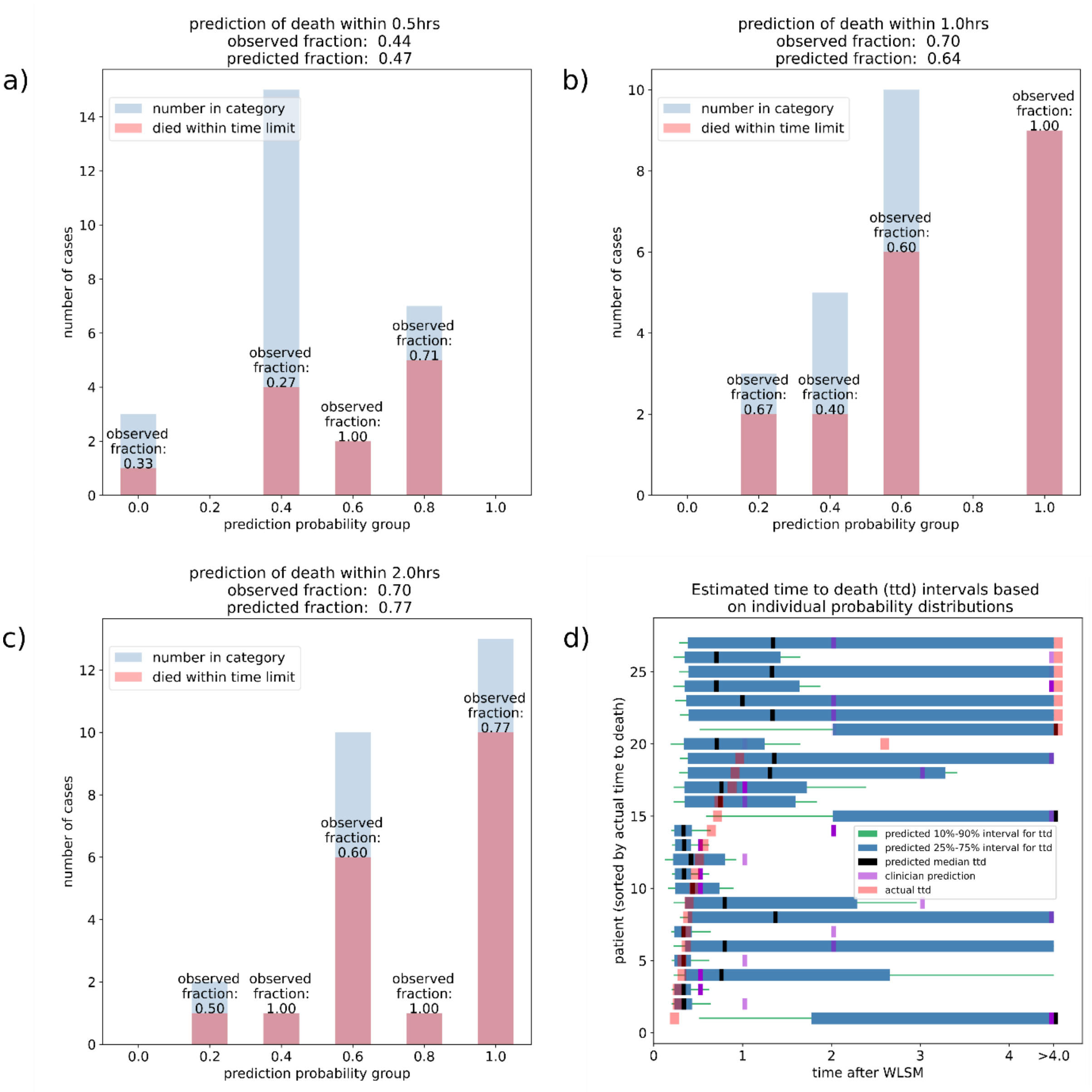

Supplementary Figure 3: Model performance in the 27 study patients, using the last prediction prior to WLSM. The individual prediction probabilities for each patient were grouped into categories, and the observed fraction of patients that died within **a)** 0.5 hours, **b)** 1 hour, or **c)** 2 hours of WLSM was calculated for each category. Due to the small number of patients, some categories had very few predictions. The overall observed fraction of death and the mean prediction probability for all patients for death within that time of interest is provided above each figure. **d)** The individual probability distributions over time for each patient are depicted here using bar plots, using interpolated probability distributions for a finer time resolution. To ease visualization, patients were sorted by their actual time to death. The predicted median time to death (ttd) is the time at which the predicted probability crosses 50%. The predicted 25%-75% interval shows the time points where the predicted probability is between 25% and 75%. By definition, 50% of patients should have a time to death that falls within this interval (the observed fraction of actual time to death values occurring in this interval is 16/27=59%). Similarly, 80% of patients should have a time to death that falls within the 10%-90% interval (the observed fraction is 23/27=85%). Note that if a patient was assigned a risk equivalent to the average observed risk of death from the derivation cohort, they would have an assigned probability of rapid death of 0.44, 0.56, 0.67, 0.71, and 0.73 at 0.5, 1, 2, 3, and 4 hours, respectively. This "average" patient would therefore have a very wide 25%-75% confidence interval (i.e. from <30 minutes to > 4 hours), as the probabilities do not cross the 25% and 75% thresholds within this time range. The

width of the 25%-75% interval can therefore be used to provide a degree of confidence for each prediction. For comparison, the clinician's prediction of time to death is shown in magenta. The darker the shading, the more confident the clinician's prediction.

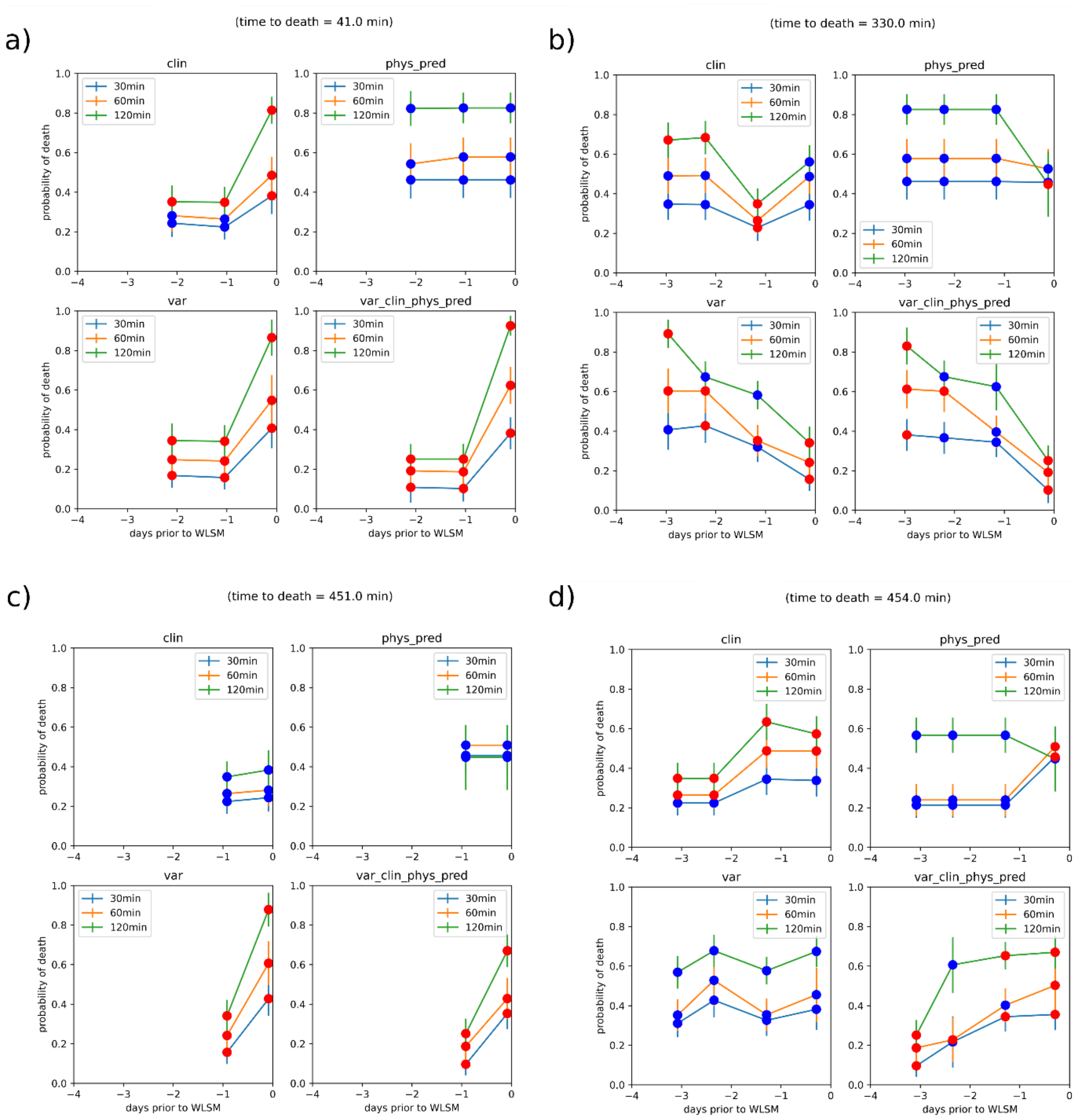

Supplementary Figure 4: Prediction probabilities over time for 4 patients with the largest probability changes for death within 2 hours using the combined model. Each figure shows predictions for the 4 different prediction models (clinical features only ("clin"), physician prediction ("phys_pred"), variability only ("var"), and the combined model ("var_clin_phys_pred"). Red circles are used to indicate probabilities that are more than 1 standard deviation from the mean prediction value over time for that patient; blue circles indicate that the prediction is within 1 standard deviation from the mean. Prediction error bars show the standard deviation for each prediction in green. The actual time to death is given above each figure. Predictions for death within 30 minutes, 60 minutes, and 120 minutes are shown for each model type. Probabilities for the physician prediction model are calculated using a random survival forest model employing the physician's prediction of time to death and their prediction confidence.
